# Toward the development of a specific non-enzymatic amperometric sensor for determining uric acid in fermentation samples

**DOI:** 10.1007/s00604-025-06979-4

**Published:** 2025-02-12

**Authors:** E. V. Butyrskaya, E. V. Zolotukhina, P. Herbeck-Engel, M. Koch, Y. E. Silina

**Affiliations:** 1 Department of Analytical Chemistry, Voronezh State University, Voronezh, Russia; 2 Federal Research Center of Problems of Chemical Physics and Medicinal Chemistry, Russian Academy of Sciences, Moscow Region, Russia; 3 INM-Leibniz Institute for New Materials, Saarbrücken, Germany; 4 HTW saar -University of Applied Sciences, Saarbrücken, Germany; 5 Department of Biochemistry, Saarland University, Saarbrücken, Germany

**Keywords:** Uric acid, Adsorption energy, Cu-NPs, Defect Cu_x_O_y_

## Abstract

This paper proposes the development of a specific non-enzymatic amperometric sensor based on electrodeposited copper nanoparticles (Cu-NPs) for the determination of uric acid (UA) in fermentation samples. Through optimization of the Cu-NPs-containing sensing layer, it was demonstrated that copper(II)-induced oxidation (catalytic effect) in the presence of molecular oxygen is more effective for determining UA than the adsorption of UA on Cu and Cu-oxide surfaces. More importantly, simply changing the sensing layer’s surface chemistry by increasing the defect Cu_x_O_y_ on the surface of Cu-NPs after heating at 70 °C for only 20 min significantly improved the specificity of UA determination in both model and real fermentation samples (viz*.* supernatants of *S. cerevisiae* and *E. coli*). This study can be used as a guideline for the future assembly of functional electrodeposited sensing layers for the specific determination of target electroactive bioanalyte(s).

**Graphical abstract:**

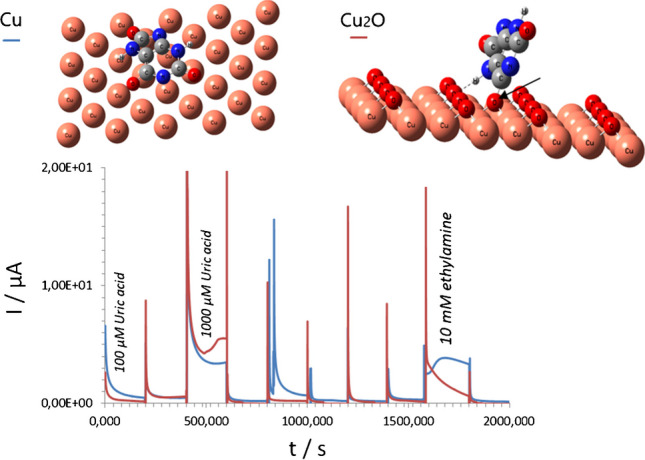

**Supplementary information:**

The online version contains supplementary material available at 10.1007/s00604-025-06979-4.

## Introduction

As a final metabolite of purine alkaloid metabolism, uric acid (UA) serves as a crucial marker of several physiological processes [1, 2]. Depending on the matrix UA concentrations can exceed 1 – 40 µM in fermentation samples [3], 0.13 – 0.56 mM in serum [4], and 1.50 – 4.50 mM in urine [5]. Therefore, the rapid, sensitive, and specific detection of UA in real samples at low concentrations is a crucial bioanalytical task [6, 7]. Conventional analytical methods, e.g., liquid chromatography or enzymatic assays with spectrophotometric or fluorometric detection applied for analysis of UA in real matrices [8, 9] require extensive sample preparation steps, including preliminary purification of samples, separation, or pre-concentration. This significantly limits the application of the conventional methods for high-throughput analysis of UA.

The enzymatic detection of UA through electrochemical read-outs is challenging due to its nature as a scavenger of oxygen radicals and its extremely high electrochemical activity on the surface of almost all known electrocatalysts [5, 7]. The biochemical oxidation of UA using enzymes (e.g., uricase) is difficult to distinguish from the parallel electrochemical reaction taking place on the surface of electrocatalysts under applied polarization [3]. In other words instead of oxidation of the product of enzymatic reaction namely the intact substrate (UA) can be oxidized on the surface of electrocatalysts by way of an electrochemical reaction that competes with the biochemical route with respect to rate, speed, and amount.

However, the use of enzyme-free electrochemical biosensors leads to pronounced matrix effects and increased signal interferences from other compounds present in samples that can be electrooxidized at the same polarization, resulting in a loss of sensitivity and selectivity during UA determination.

In contrast to selectivity (the ability to determine a particular bioanalyte within a complex mixture without signal interference from other components) specificity refers to the ability to determine the presence of a single analyte within a matrix regardless of its type [6, 10, 11].

Several sophisticated approaches have been proposed to improve the selectivity and specificity of UA analysis in the absence of enzymes, for example*,* by modifying/engineering nanozymes by introducing recognized sites (molecularly imprinted polymers, MIPs) [12–14], through the separate and simultaneous determination of UA, xanthine, and hypoxanthine using resolved current responses under corresponding applied potential [15, 16], and by engineering a single-atom catalyst to build an electrochemical biomimetic sensor for UA detection [17].

However, tuning the surface chemistry composition of the functional sensing layer of electrodes may be a simpler, faster, and more reliable alternative. We can thus hypothesize that the specificity and selectivity of electrodes with sensing layers are closely linked to the analyte’s adsorption stage. The binding efficiency and concentration of the electroactive form(s) of target analyte(s) at the near-electrode surface are impacted by their adsorption energy. Therefore, controlled modifications of the surface chemistry of functional layers of electrodes can lead to changes in target analyte(s)’ adsorption energies, thus changing the sensor’s detection sensitivity, selectivity, and specificity. In other words, controlling the surface chemistry of sensing layers of electrodes may facilitate the adjustment of their electroanalytical performance.

To verify this hypothesis, the properties of electrodes modified by intact electrodeposited copper nanoparticles (Cu-NPs) and after their controlled oxidation were studied in both model UA and fermentation solutions (e.g., supernatants obtained from *S. cerevisiae* and *E.coli*).

Our findings revealed that the specificity of UA determination using Cu-NPs-modified electrodes indeed appears to be a function of their surface chemistry. However, in contrast to our expectations, the adsorption of UA on the surface of Cu-NPs-modified electrodes was not established as a key factor influencing the specificity of UA determination. Instead, the catalytic activity of copper ions together with the presence of oxygen groups on the electrode surface plays a major role. Briefly, the controlled oxidation of Cu-NPs-modified electrodes through heating in air at 70 °C only for 20 min increases the amount of a defect Cu_2_O_x_ (*x* ≤ 1) on the surface enhancing the copper(II)-induced oxidation (catalytic effect) of urate ion at pH 9 and the formation of [Cu^+^(UA^−^)] and [Cu^2+^(UA^−^)_n_] complexes.

## Experimental part

### Chemicals and materials

CuSO_4_, uric acid (UA), KOH (pellets), phosphate salts for buffer preparation, ascorbic acid (AA), ethylamine, ethanolamine, EtOH, glycerol, urea, yeast fermentation HC complete medium (Hartwell’s Complete, supplemented with 2% glucose) and M9 *E. coli* medium (2% glucose used as a carbon source), derivatization agent N-Methyl-N-(Trimethylsilyl)-trifluoroacetamide (activated with ethanethiol and ammonium iodide), MSTFA, were received from Merck (Darmstadt, Germany). Oxygen-free standard solution (OXCAL for deoxygenation, 0% O_2_) was obtained from (Pyro Science GmbH, Aachen, Germany). An Amplex™ Red UA Kit was obtained from Thermo Fisher Scientific (Waltham, MA, USA).

Screen printed electrodes (SPEs) produced on ceramic substrates (substrate dimensions: length 3.38 cm, width 1.02 cm, thickness 0.05 cm) and modified by graphene oxide (GO, DPR-110RGPHOX), reduced graphene oxide (r-GO, DPR-110GPHOX) and carbon (DRP-110) were purchased from DropSens (Metrohm, Germany). The diameter of the working electrodes was 0.4 cm, the reference electrode material was silver, and the auxiliary electrode material was carbon.

### Formation of Cu-NPs-based sensing layers

At alkaline pH UA might be effectively oxidized by oxygen in the presence of catalytic amounts of copper [3, 18]. Various studies have reported that different structures, sizes, and forms of copper, e.g., copper (I) and copper (II) species, copper-oxides, and their amounts and ratios support the electrooxidation of UA in various concentration ranges [19–21].

For this study, we synthesized several types of Cu-NPs using electrodeposition on SPE surfaces to unify and systematize the impact of their size, morphology, and chemistry on the basis of the same synthesis platform. Cu-NPs were electrodeposited on the surface of SPE/graphene oxide (GO) at -0.5 mA for 30 s from 100 and 30 mM CuSO_4_ electrolytes (containing Cu^2+^).[3] This procedure was repeated three times to increase the amount of Cu-NPs on the surface of the electrodes. To study the impact of the nature of the electrode support substrate on Cu-NPs’ amperometric response to UA, the electrodeposition procedure was also conducted on SPEs modified by reduced graphene oxide (r-GO) and those modified by carbon. After the formation of the sensing Cu-NPs-based layer the electrodes were washed with DI water.

### Electrochemical studies

A one-channel PalmSens4 potentiostat (PalmSens, Utrecht, The Netherlands) in cyclic voltammetry (CV) mode at a scan rate of 50 mV/s was used to evaluate the electrochemical performance of SPEs with electrodeposited Cu-NPs in a 150-µL droplet of UA model solutions or cell supernatants (see below) [3]. CV was also recorded in UA dissolved in the oxygen-free solution to evaluate the impact of oxygen present in the droplet and to verify the influence on UA oxidation of the oxygen in the electrode material. The specificity of the produced Cu-NPs-modified electrodes *vs.* silver oxide electrodes was assessed in amperometric (AM) mode at 0.22 V.

The electrodes were calibrated in a multi-step amperometric mode (MAM). Briefly, this involved level 1 polarization at -0.1 V held for 60 s, followed by polarization at level 2 at 0.22 V for 30 s [3]. Recoveries of UA in samples were determined using the external standard approach (ESTD) at pH 9. The quantitative analysis of UA in real fermentation samples was conducted using the multiple standard addition approach by adding known quantities of the target analyte to the sample [22, 23].

### Measurement of oxygen in a droplet

To evaluate the droplet’s oxygen content in phosphate buffer and oxygen-free solutions (see above) in the presence and in the absence of UA, an OXR430 needle oxygen minisensor (PyroScience GmbH, Aachen, Germany) was used to measure the oxygen concentration (µmol·L^−1^). The needle of the oxygen minisensor was placed into the droplet of the test solution directly on the surface of Cu-NPs-modified SPEs. The signal read-outs were conducted while simultaneously starting CV.

### Cell cultivation and obtaining of real samples containing UA

For determination of UA in supernatants of *E. coli* at their physiological significant points, the cultivation of cells was conducted in 50 mL flasks in M9 medium at 37 °C for 4, 7, and 12 h [24]. Cultivation of yeast cells for visualization of UA amount in their supernatants requires a longer time [3]. Therefore, yeast cells (*S. cerevisiae,* BY4742) were cultivated in 50 mL flasks in HC medium at 32 °C for 70 h and 96 h. The optical density (OD) of the obtained cell suspension was measured on an Ultrospec 10 instrument (Thermo Fisher Scientific). Afterward, cells were removed from the suspension by centrifugation at 13,000 rpm for 10 min. The obtained supernatants were used in the subsequent electrochemical experiments. Prior to investigations the pH of the supernatants was adjusted to 9 ± 0.02.

### Gas-chromatography mass spectrometry (GC–MS)

For the analysis of the content of the received supernatants, e.g., amines the received samples were derivatized with 40 µL of a MSTFA at 60 °C for 30 min [23]. Next, 1 µL of the received sample was injected into the GC MS system (TSQ-9000 Trace 1310, triple quadrupole MS, Thermo Fisher Scientific), equipped with an autosampler, Thermo TriPlus).

GC – MS separation was carried out on a MXT column (cross-bond 100% dimethyl polysiloxane, 0.25 mm × 30 mm × 0.25 µm thickness) in the splitless mode under the following gradient conditions: starting temperature 100 °C held for 2 min, raised to 220 °C at 20 °C/min for 7 min and held at 300 °C for 13 min. The mass spectra were recorded in both TIC modes in the range of *m/z* 100 500. MS transfer line temperature was 250 °C.

### KIT for analysis of uric acid in real samples (Uric Acid/Uricase KIT)

To determine UA in the supernatants of tested cells, an Amplex™ Red Uric Acid/Uricase KIT assay (Thermo Fisher Scientific) was used. Measurements were conducted at 585 nm in fluorescence microplates on an FP-8500 spectrofluorometer (Jasco, Tokyo, Japan) according to a previously reported protocol [3].

### Scanning electron (SEM) and energy dispersive X-ray (EDX) studies

For SEM analysis of Cu-NPs-modified electrodes, an FEI (Hillsboro, OR, USA) Quanta 400 FEG was used in high vacuum mode. Secondary and backscattered electrons (BSE) were detected at 10 kV accelerating voltage using the Everhart-Thornley/Solid-State-Detector. X-ray spectroscopic analysis was performed using an EDAX (Wiesbaden, Germany) Genesis V6.04 spectrometer.

### RAMAN spectroscopy studies

A Raman microscope LabRAM HR Evolution (HORIBA) equipped with a 633-nm HeNe laser was used to investigate Cu-NPs-modified SPEs. The G band of the carbon matrix was scaled to provide a rough estimate of the increased formation of copper oxides after heating to 70 °C.

### Quantum-chemical calculations

At pH 9 UA is present in solutions as a form of urate anion [25]. Quantum-chemical calculations were hence performed for the urate anion located on representative fragments of Cu, Cu_2_O, and CuO crystals. Model structures were optimized and adsorption Gibbs energies were calculated using the density functional theory (DFT) B3LYP/6-31G(d,p) [30]. The influence of solvent was taken into account in the polarizable continuum model (PCM). The sorbent atoms were frozen during the optimization process, and only the position of the urate anion was optimized. The GaussView program was used to visualize the optimized structures. The applied DFT function enables the visualization of molecular interactions between the urate ion and Cu surfaces [26, 27]. To compare the specificity of Cu-NPs-modified electrodes to UA, the total Gibbs adsorption energies (*G*_ads_) were also calculated for interfering compounds, viz*.* ethylamine.

## Results and discussion

### Design and characterization of electrodes modified with electrodeposited Cu-NPs

**Fig. 1:**
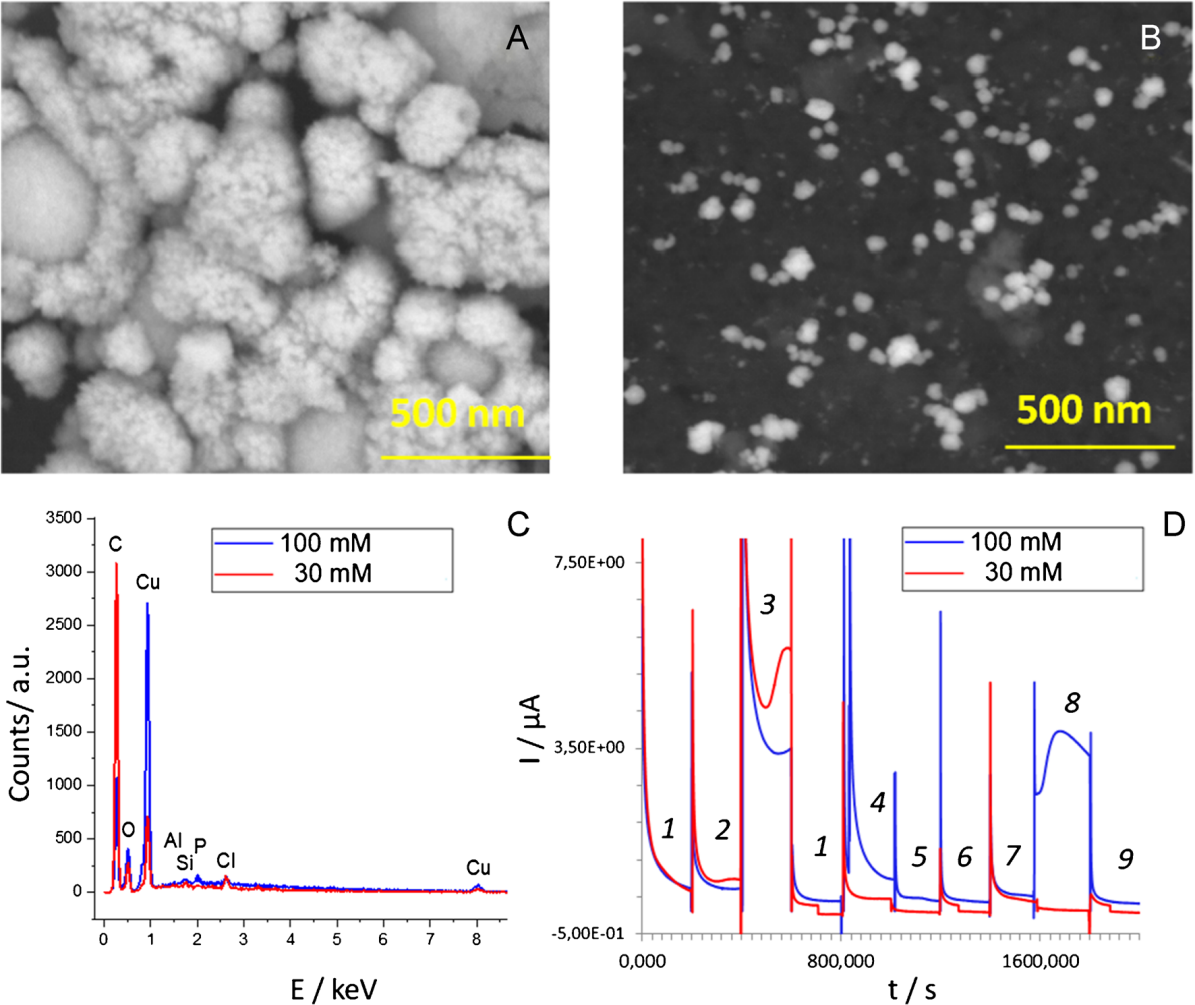
BSE images (**A**, **B**), EDX spectra (**C**), and AM plots (**D**) were recorded at 0.22 V from Cu-NPs-based electrodes prepared from 100 mM (**A**) and 30 mM (**B**) copper-containing precursor solutions. In **D** the performance of both electrodes was tested in a droplet of *1 - *buffer; *2 - *100 µM UA, *3 - *1 mM UA, *4 - *100 µM AA (ascorbic acid), *5 - *10 mM glycerol; *6 - *10 mM ethanol, *7 - *10 mM ethylamine; *8 *- 10 mM ethanolamine, *9 - *10 mM urea. *Note*: the pH of all tested solutions was 9 ± 0.2

Depending on the experimental setup a nearly dense copper layer or copper islands in the range of 80 – 120 nm were deposited on GO/SPE at -0.5 mA from 100 mM and 30 – 60 nm from 30 mM electrolyte solutions, respectively (Fig. 1A, B). The copper precipitates electrodeposited from 100 mM electrolyte consisted of smaller nanoparticles with a size of 10 – 20 nm. EDX analysis revealed the presence of oxygen on the surface of both electrodes which could equally be attributed either to graphene oxide (GO) or copper oxides formed during electrodeposition (Fig. 1C).

The electrodes produced from 30 mM electrolyte (Fig. 1B), despite the lower copper content on their surface, demonstrated an advanced electroanalytical performance compared with analogs produced from 100 mM electrolyte (Fig. 1D). The electrodes modified with small Cu-NPs thus mostly responded to UA with a less pronounced signal for amines. In contrast, the electrodes modified with larger Cu-NPs (produced from 100 mM electrolyte) demonstrated significant sensitivity to amines (shown for ethylamine and ethanolamine as a case study), with current intensities almost comparable to those with UA.

It should be mentioned, that the copper-catalyzed aerobic oxidation of amines and amino-group containing compounds is well-known [28–31]. However, real fermentation samples contain a wide spectrum of amines (e.g., ~ 5 – 7% of the total amount of detected compounds (ESI, Fig. S1, and Table S1), shown for supernatants of yeast cells as an example). This means that using Cu-NPs-modified electrodes to analyze UA concentrations in real fermentation samples can result in an additive signal that corresponds to the oxidation of both compounds (e.g., amines and UA) and false quantitative results of the target analyte (UA).

Taking into account the low UA content found using Amplex™ Red Uric Acid/Uricase KIT in the tested fermentation samples (ESI, Table S2), making further improvements to the specificity of Cu-NPs-modified electrodes is highly desirable (see following sections).

### Impact of the nature of electrode support on the amperometric behavior of Cu-NPs-modified electrodes in UA electrooxidation in oxygenated alkaline solution

**Fig. 2:**
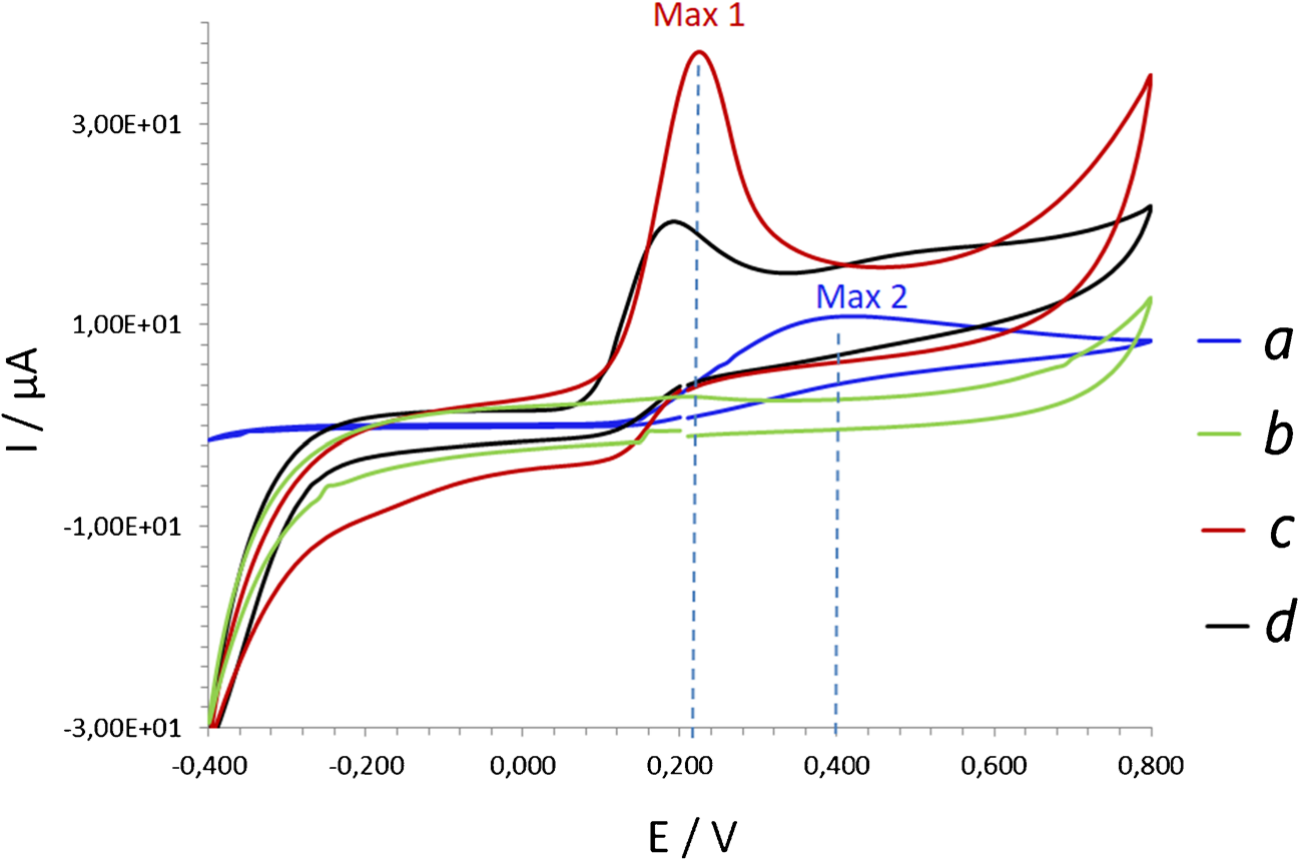
CV plots recorded at 50 mV/s from pure GO/SPE in 1 mM UA (**a**); GO/SPE modified with small Cu-NPs (30 mM electrolyte) tested in buffer (**b**) and in 1 mM UA (**c**); GO/SPE modified with large Cu-NPs (100 mM electrolyte) tested in 1 mM UA (**d**), *Note:* the pH of tested solutions was 9 ± 0.2

The electroanalytical performance of Cu-NPs-modified electrodes was evaluated in CV mode in model UA solutions at ambient conditions (in the presence of a dissolved oxygen), (Fig. 2). A pure GO/SPE was used as a reference in these experiments. UA was oxidized on the surface of GO/SPE with the peak maximum seen at 0.4 V, (Fig. 2a). In contrast, on the surface of GO/SPE modified with Cu-NPs UA was oxidized at 0.2 V, regardless of the size of nanoparticles (Fig. 2c, d). Notably, electrodes modified by smaller Cu-NPs (Fig. 2c) demonstrated an advanced electroanalytical performance compared with the larger Cu-NPs analog (Fig. 2d).

Besides the electrochemical conditions and, pretreatment of the sample or electrode surface, the influence of the electrode support material on the recorded analytical signal cannot be excluded [10]. This assumption was verified through specificity tests utilizing electrodes modified with small Cu-NPs (30 mM electrolyte) electrodeposited on a commercial carbon electrode, GO/SPE, and reduced GO, r-GO/SPE. All tested electrodes had the same geometric area and the electrodeposition was carried out at the same polarization mode (see Experiment, Section Formation of Cu-NPs-based sensing layers).

Remarkably, the responses of tested electrodes with the electrodeposited Cu-NPs to even a minor concentration (100 µM) of UA were almost identical, regardless of the material support (ESI, Fig. S2A). This suggests that the electrode support material does not significantly influence the electrochemical behavior of Cu-NPs-electrocatalysts in model UA solutions in the presence of molecular oxygen in a droplet.

However, it appears to be possible to alter the electroanalytical performance of Cu-NPs-modified electrodes with a short temperature treatment (e.g., 70 °C on the air for 20 min). Briefly, after thermal treatment of Cu-NPs-based electrodes, the detected response to amines was much less pronounced for all tested systems, regardless of the electrode support material used (viz*.* carbon, GO or r-GO) (ESI, Fig. S2B – D). The same result was observed for the electrodes modified with larger Cu-NPs after being heated at 70 °C (ESI, Fig. S3A).

Notably, after heating at 70 °C no changes in the morphology of Cu-NPs can be expected (SEM/BSE images also did not reveal any pronounced changes) indicating the preservation of the electroactive surface area (ESCA) of electrodeposited Cu-NPs and stability of the sensing layer (ESI, Fig. S3B, C). If the impact of morphological changes and ESCA can be excluded, then the improvements in the specificity of UA detection by synthesized electrodes in the presence of dissolved oxygen after heating can only be related to changes in the surface chemistry of Cu-NPs.

It is hypothesized that changes in the surface chemistry of tested electrodes that maintain the same electroactive surface area can result in changes in the adsorption energies of the target analyte, e.g., UA and amines [22, 32] Additionally, changes in the Gibbs adsorption energies (*G*_ads_) result in changes in the electroanalytical performance of electrodes modified with the same electrodeposited Cu-NPs. This hypothesis was confirmed during the subsequent RAMAN studies and quantum chemical calculations (see next sections).

### Mechanistic aspects underlying the advanced electroanalytical performance of thermally treated electrodes modified with Cu-NPs

Based on the observed dependencies, we can assume that copper oxides form on the surface of electrodeposited Cu-NPs. Indeed, using Cu_2_O and CuO in the design of functional layers of non-enzymatic electrochemical sensors for the advanced detection of UA in various mediums and with varying analytical merits has previously been explored [19, 33, 34].

**Fig. 3:**
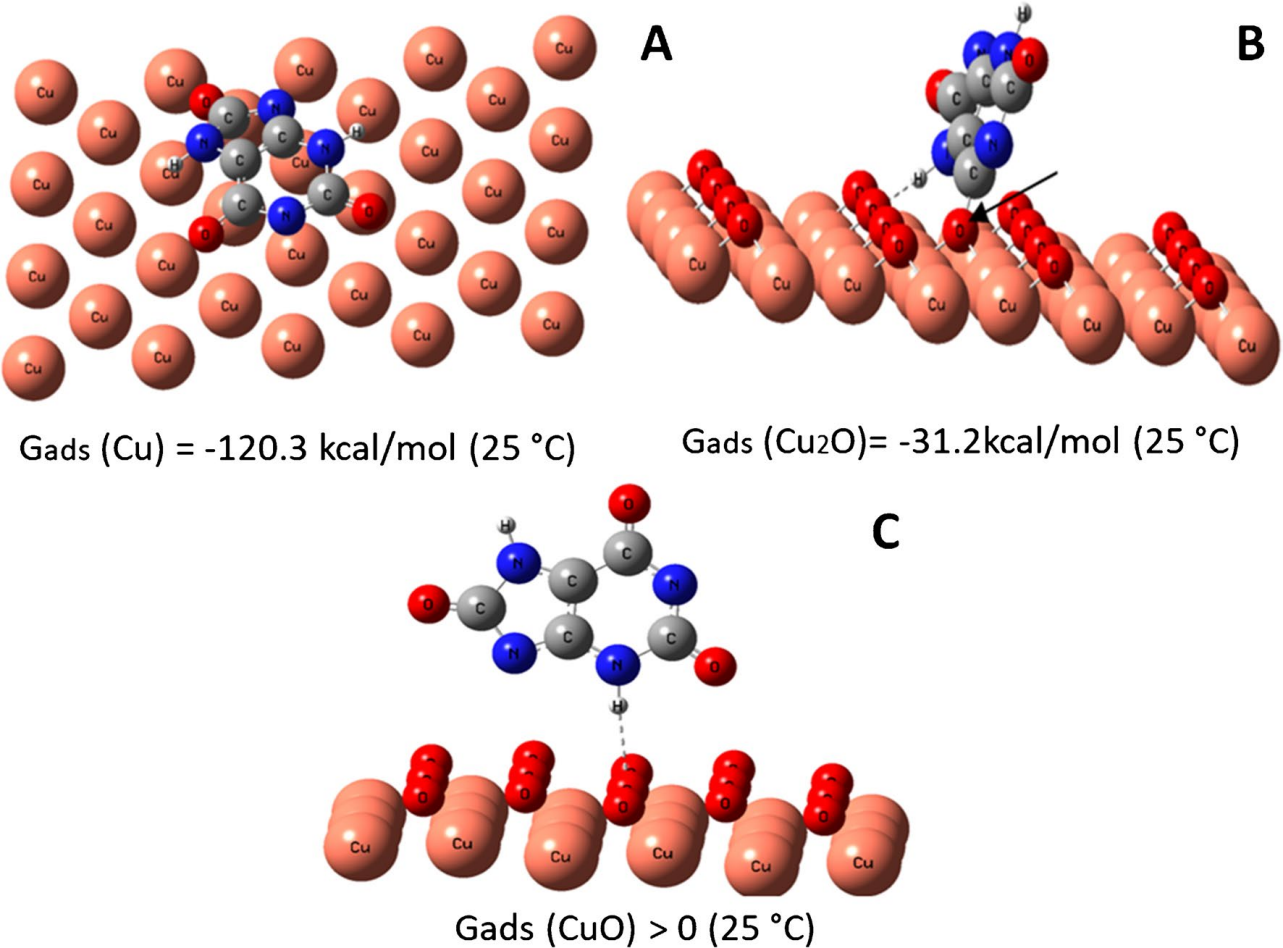
The Gibbs adsorption energies (*G*_ads_) and the most favorable location of urate ion on Cu (**A**), Cu_2_O (**B**), and CuO (**C**) surfaces. *Note*: the dashed line represents the hydrogen bond; the arrow indicates the donor-acceptor bond between the oxygen of UA and copper atoms

At the same time, our quantum-chemical calculations indicated reduced adsorption energy (*G*_ads_) of the urate ion (at pH 9 UA exists in the form of the urate ion) on the surface of copper oxides (e.g., Cu_2_O and CuO) *vs* Cu (Fig. 3).

The adsorption energy of the urate ion decreases from -120.3 kcal/mol to 0 kcal/mol in the line of Cu → Cu_2_O → CuO. The high adsorption energy on Cu is because the sorbate rings are arranged parallel to the sorbent surface (Fig. 3A), and all the O- and N- atoms of urate are near the surface. Due to the presence of a free electron pair, these atoms form a large number of donor–acceptor bonds with the Cu surface.

On the Cu_2_O surface, UA is anchored by two donor–acceptor bonds and one hydrogen bond (Fig. 3B), therefore the adsorption energy is reduced compared with Cu. In contrast, on the CuO surface, the urate ion is fixed by a single hydrogen bond insufficient to keep it on the surface. This means that the urate anion can readily be desorbed from the surface of CuO into the droplet/solution, where it forms H-bonds with water molecules.

In addition, the adsorption energy of ethylamine (analyte interfering in the detection of UA by Cu-NPs, Fig. 1D) in the same line/process of Cu → Cu_2_O → CuO remains almost at the same level, ESI, Fig. S4. This cannot altogether explain the advanced electroanalytical performance of electrodes modified with Cu-NPs, viz*.* improvements in the specificity of electrodes toward UA detection (e.g., reduced impact of amines) after being heated at 70 °C in air.

In fact, Cu_2_O and CuO can rarely be detected on the surface of thin copper-containing films. In ambient conditions, copper reacts with oxygen to form non-stoichiometric copper oxides [35].

RAMAN studies were conducted to verify the type(s) of oxides formed on the surface of Cu-NPs during direct electrodeposition and after electrode heating at 70 °C. Regardless of the electrode material used as a template for the electrodeposition of Cu-NPs, e.g., carbon, r-GO, or GO, the formation of a defect copper oxide (II) on the surface of the electrodeposited Cu-NPs was revealed (ESI, Fig. S5).

More specifically, the best agreement between RAMAN spectra recorded from the electrodeposited Cu-NPs and reported in the literature data for copper films and nanoparticles corresponded to the formation of defective copper oxides (Table S3). Briefly, the bands at < 220 cm^−1^, 528 cm^−1^, and 612 cm^−1^ with a shoulder at 638 cm^−1^ indicate the formation of primarily defective Cu_2_O formed on SPE/GO and SPE/carbon. In contrast, the band at < 220 cm^−1^ is missing on the SPE/r-GO surface. The appearance of two bands at 530 cm^−1^ and 600 cm^−1^ indicates the formation of Cu_4_O_3._

However, the same types of oxides (a mixture of Cu_2_O, Cu_4_O_3_, and CuO), at higher amounts were detected for Cu-NPs-based electrodes after heating at 70 °C. This result aligned with measurements reported for in situ copper oxidation carried out in the air in the range of 70 – 130 °C [36]. A further temperature increase to 150 – 200 °C and 250 °C results in the formation of Cu_3_O_2_ and CuO, respectively. In other words, the 70 °C thermal treatment of the studied Cu-NPs-modified electrodes increases the amount of the defect Cu_x_O_y_ (ESI, Fig. S5A *vs* Fig. S5B).

It should also be emphasized, that in the defect copper oxide, numerous vacancies, interstitials, or antisite defects can be observed [35]. This suggests that Cu^+^ and Cu^2+^ can readily be available on the surface of Cu-NPs-modified electrodes to interact with the urate ion at alkaline pH.

As stated in the literature [17, 20, 26], Cu^2+^ ions facilitate UA oxidation in the presence of molecular oxygen. During electrooxidation urate/copper complexes can be formed, e.g., [Cu^+^(UA^−^)], [Cu^2+^(UA^−^)n]. It is possible, that copper ions present under anodic polarization on the surface of Cu-NPs and their defect oxide structures play the same catalytic role. In other words, copper (II)-induced oxidation of urate results in the formation of copper-urate complexes (the activation energy copper-uric acid-O_2_ is about 16.950 cal per mole [20] indicating the formation of a relatively stable complex) which may explain the advanced performance of Cu-NPs-based electrodes treated at 70 °C.

To summarize, thermal treatment of Cu-NPs-modified electrodes increases the amount of defect copper oxides (ESI, Fig. S6) that facilitates copper(II)-induced oxidation of the urate and formation of [Cu^+^(UA^−^)] and [Cu^2+^(UA^−^)_n_] complexes. In terms of electrocatalysis it can be concluded that a Cu-surface has fewer electrocatalytic properties to UA oxidation *vs* Cu^2+^ contained in the defect structure of copper oxides. In other words, copper(II)-induced oxidation (catalytic effect) in the presence of molecular oxygen is more effective for determining UA than the adsorption of UA on Cu and Cu-oxide surfaces.

With respect to a future nanoengineering strategy, the obtained results mean that increasing the number of Cu(II) centers available on the surface of electrodeposited Cu-NPs for the adsorption of UA will increase the UA rings that can be located parallel to the surface of Cu_2_O_x_ above the copper section, in turn enhancing the potential sensitivity and specificity of the analysis.

### Role of oxygenated groups of material support in the response of Cu-NPs-based electrodes at alkaline pH

Several studies [18, 20, 25] have emphasized the rope of molecular oxygen in the electrooxidation mechanism of UA on copper surfaces resulting in the formation of stable [Cu^+^(UA^−^)] and [Cu^2+^(UA^−^)_n_] complexes. In Section Impact of the nature of electrode support on the amperometric behavior of Cu-NPs-modified electrodes in UA electrooxidation in oxygenated alkaline solution, it was shown that the material support of electrodes had almost no influence on the efficiency of UA electrooxidation in the presence of dissolved molecular oxygen. However, how the electroanalytical performance of electrodes with electrodeposited Cu-NPs at alkaline pH can be influenced by the absence of dissolved oxygen in both model and fermentation mediums remains unclear.

**Fig. 4:**
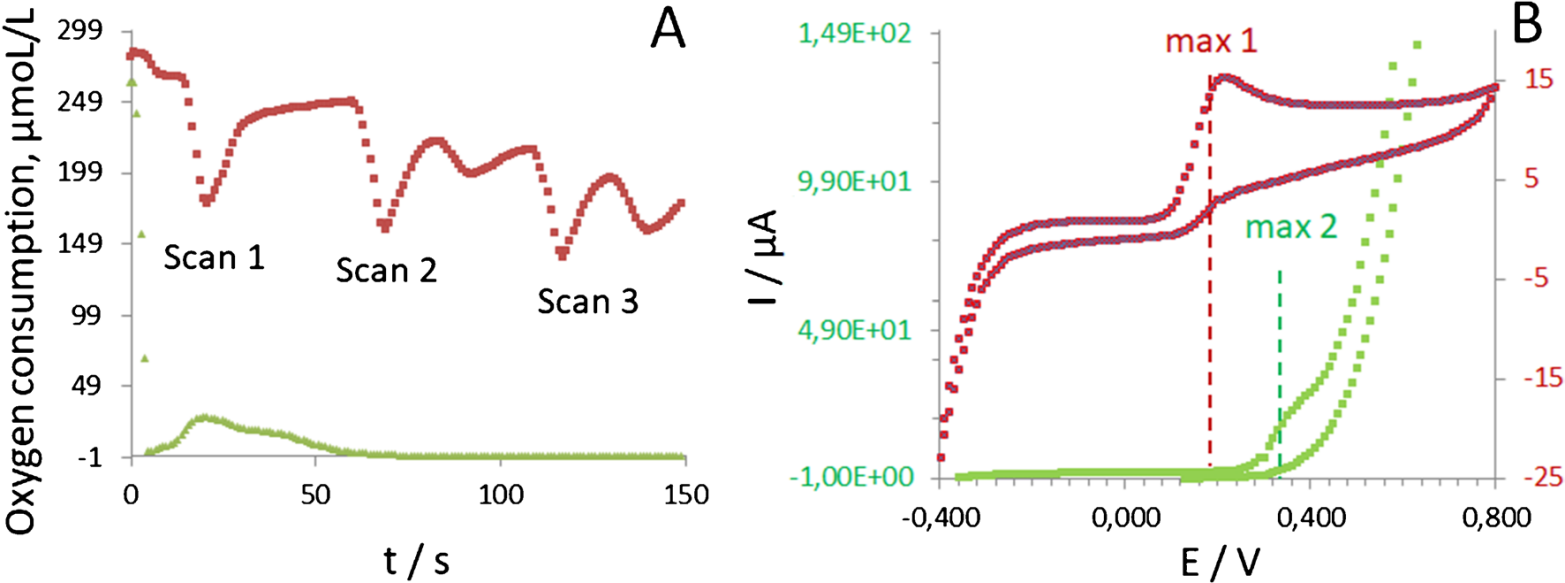
Dynamic oxygen mini-sensor response (**A**) and CV plots (**B**) recorded at 50 mV/s from Cu-NPs-modified GO/SPE in CV mode in 1 mM of UA prepared in a conventional phosphate buffer (red line) and in deoxygenated buffer (green line). *Note*: in (**B**) 2d scans shown; pH of test solutions was 9 ± 0.2

To address this question, UA was dissolved in oxygen-free deoxygenated buffer (OXAL) maintaining the same alkaline conditions. Surprisingly, even in the absence of the dissolved oxygen (Fig. 4A), the response of Cu-NPs-modified electrodes to UA could still be recorded (Fig. 4B), e.g., with a shift from 0.22 V (in the presence of oxygen) to 0.35 V (in the absence of oxygen).

This experiment demonstrated that the oxidation of UA on Cu-NPs-modified electrodes may occur even in the absence of dissolved oxygen in a droplet of test solution. At the same time, the efficient oxidation of an analyte cannot take place in the absolute absence of oxygen [5, 7]. Therefore, it can be assumed that the electrode support material has an influence and supports the electro-oxidation of UA at alkaline pH in the absence of dissolved oxygen.

This assumption was confirmed by subsequent model experiments in an oxygen-free solution containing UA. The electrooxidation of UA in oxygen-free solutions was studied on pure GO, r-GO, and carbon SPEs to achieve this objective. The obtained results were compared with those recorded from the same electrodes modified by electrodeposited Cu-NPs (30 mM electrolyte).

**Fig. 5:**
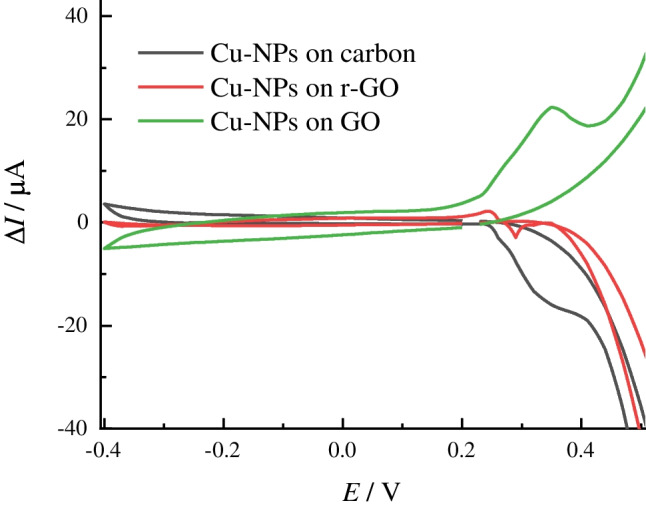
Current transients obtained in CV at 50 mV/s for Cu-NPs modified SPEs of different natures (see legend) in 1 mM UA solution in deoxygenated solution at pH 9. *Note:* current responses in the same deoxygenated solution were subtracted

The subtraction of anodic currents recorded in a deoxygenated buffer in both the absence and presence of UA indicates that the oxygen species of electrode material support may indeed participate in the oxidation of UA in the absence of dissolved oxygen in a droplet (Fig. 5).

It can be observed from Fig. 5 that after subtracting of currents obtained from copper-modified SPEs in UA oxygenated solution and the currents recorded in the same solution from unmodified (pure) electrodes, only the electrode with the Cu-NPs deposited on the surface of GO/SPE exhibits electrocatalytic activity to UA in the absence of oxygen (Fig. 5, green line). The other tested electrode material supports (e.g., carbon and r-GO) remain catalytically non-active in an oxygen-free environment after modification with the electrodeposited Cu-NPs (Fig. 5, black and red lines).

Based on the obtained data it can be concluded that for the enhanced copper (II)-UA complexes formation the electrode material containing oxygenated groups, e.g., GO supports the oxygen supply/transfer providing an effective electrooxidation of UA at alkaline pH even if the medium lacks oxygen.

**Fig. 6:**
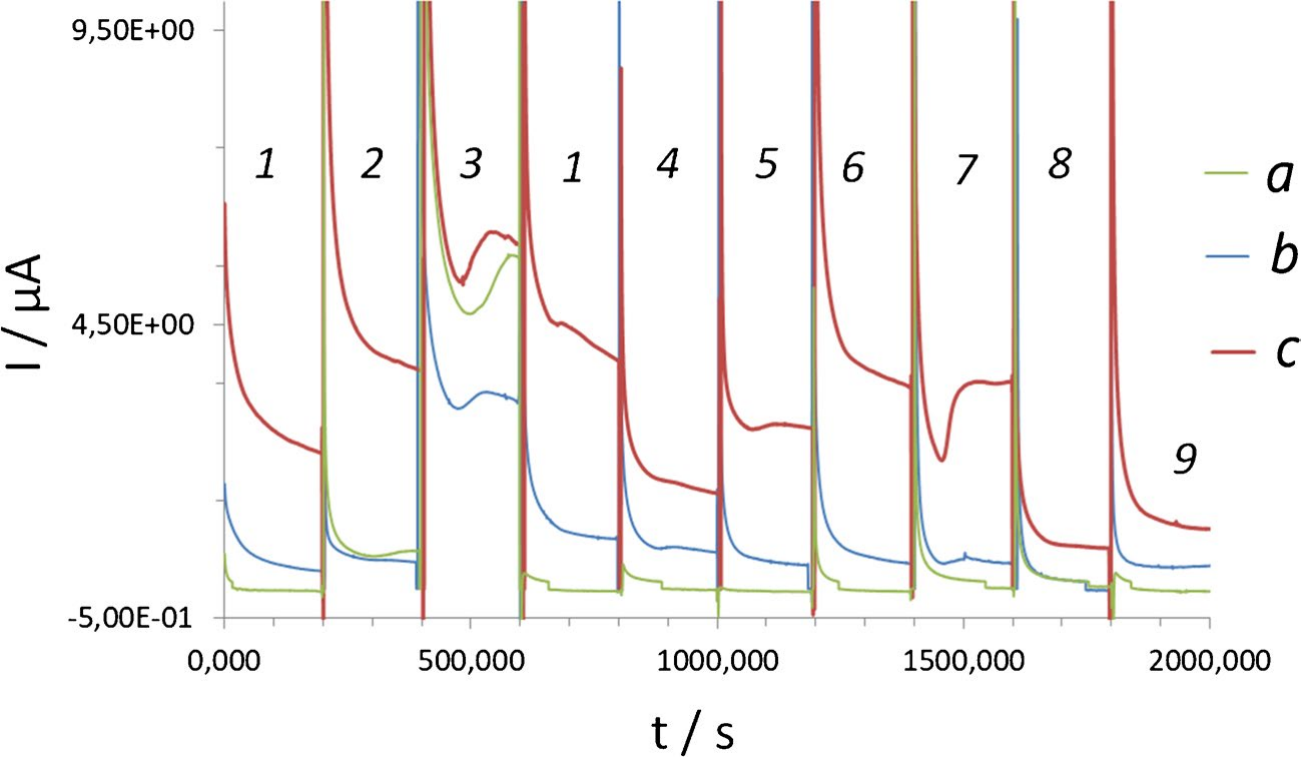
AM plots recorded at 0.22 V (**a**,**b**) and 0.35 V (**c**) from Cu-NPs-modified GO/SPE heated at 70 °C and contained defect Cu_2_O_x_ (**a**) tested in the presence of oxygen in: 1 - buffer; 2 - 100 µM UA, 3 - 1 mM UA, 4 - 100 µM AA, 5 - 10 mM glycerol; 6 - 10 mM EtOH, 7 - 10 mM ethylamine; 8 - 10 mM ethanolamine, 9 - 10 mM urea. **b**,**c** Same electrode tested in the same analytes in OXCAL deoxygenated buffer solution containing Na_2_SO_3_. *Note*: the pH 9 of all tested solutions was 9 ± 0.2

It should also be emphasized that the oxygen-free buffer OXCAL contains a reducing Na_2_SO_3_ compound. Applying this buffer to the electrodes modified with the electrodeposited Cu-NPs leads to the reduction of the copper oxide defects and a significant loss in the specificity of UA detection regardless of the applied potential (Fig. 6) (comparison with Cu-NPs-modified electrodes containing copper oxide defect, e.g., Cu_2_O_x_, no oxygen-free buffer containing Na_2_SO_3_ used, line a). Once copper oxide defects reduce (lines b,c), almost no specificity to UA can be obtained from the same electrode. These results confirm the importance of the presence of copper oxide defects on the surface of Cu-NPs for the specificity of electrodes to UA detection.

### Application of Cu-NPs-based electrodes to UA sensing in fermentation samples

A lack of dissolved oxygen at alkaline pH has been previously reported in real fermentation medium solutions [32]. Therefore, it was important to explore the behavior of Cu-NPs-modified GO/SPE in fermentation samples.

ESTD was used to define the linear dynamic range (LDR) and limit of quantification (LOQ) for UA in model solutions by GO/SPEs modified with small Cu-NPs (30 mM electrolyte) and larger Cu-NPs (produced from 100 mM electrolyte) between 2 and 100 µM, and 5 and 50 µM (LDRs), and 2 µM and 5 µM (LOQ), respectively. Because of the advanced analytical merit obtained from electrodes modified with small Cu-NPs (produced from 30 mM electrolyte) these systems were further used for the determination of recoveries and analysis of real samples. It should also be mentioned that the determination of UA by intact Cu-NPs-modified electrodes utilizing the ESTD approach led to false and significantly inflated results regardless of the type of the matrix used, e.g., yeasts or E*. coli* supernatants, (Table 1).

**Table 1:** Selected UA recoveries in supernatants of cells recorded by ESTD at pH 9 ± 0.2 in MAM mode (read-out at 0.22 V) from GO/SPE modified with Cu-NPs

Sensing layer	Calibration formula, *R*^2^	Added UA, µM	Found UA, ± SD, µM	Recovery, %	RSD, %
OD = 6.1, yeast cells were cultivated for 96 h, UA amount found in the supernatant by KIT is 0.79 µM					
Cu-NPs/GO (original)	*y* = 0.006*x* + 0.035 *R*^2^ = 0.9969	5.00	15.86 ± 0.121	*Non-acceptable*	7.7
		10.00	19.86 ± 0.010		5.4
		100.00	120.86 ± 0.033		3.7
Cu_2_O_x_-NPs/GO (heated)	*y* = 0.0046*x* + 0.099 *R*^2^ = 0.9974	5.00	5.20 ± 0.02	104	1.04
		10.00	11.50 ± 0.01	115	9.09
		100.00	80.60 ± 0.01	80.6	27.6
OD = 6.32, *E. coli* cells were cultivated for 12 h, UA content found in the supernatant by KIT is 0.62 µM					
Cu-NPs/GO (original)	*y* = 0.006*x* + 0.035 *R*^2^ = 0.9969	5.00	15.29 ± 0.005	*Non-acceptable*	3.26
		10.00	7.50 ± 0.005		4.68
		100.00	61.47 ± 0.006		1.17
Cu_2_O_x_-NPs/GO (heated)	*y* = 0.0046*x* + 0.099 *R*^2^ = 0.9974	5.00	4.47 ± 0.001	89.4	7.70
		10.00	9.57 ± 0.008	95.7	5.41
		100.00	91.50 ± 0.061	91.5	3.70

However, after thermal treatment of the same electrode at 70 °C for 20 min the recovery values ranged from 80 to 115%.

To exclude the influence of the matrix composition on the quantitative analysis of real samples, the UA in supernatants was determined using the multiple standard addition approach [4]. This approach enables accurate target analyte quantification in complex matrices and the compensation of matrix interferences. The results are summarized in Table 2.

**Table 2:** Selected UA quantitative results in supernatants of target cells found at pH 9 ± 0.2 using GO/SPE modified with Cu-NPs

Sensing layer	Calibration formula	*R*^2^	Concentration of UA, µM	RSD, %
Yeasts, OD = 6.1, cells were cultivated for 96 h, UA concentration found by KIT is 0.79 µM				
Cu-NPs/GO (original)	*y* = 0.220*x* + 66	0.9769	4.54 ± 0.10	8.7
Cu_2_O_x_-NPs/GO (heated)	*y* = 1.152*x* + 530	0.9968	0.86 ± 0.04	3.7
*E.coli* supernatant, OD = 3.8, *E. coli* cells were cultivated for 7 h, UA concentration found by KIT is 3.29 µM				
Cu-NPs/GO (original)	*y* = 1.935 *x* + 97	0.9659	7.35 ± 0.30	9.5
Cu_2_O_x_-NPs/GO (heated)	*y* = 1.321*x* + 69	0.9689	3.03 ± 0.01	2.2

The repeatability of the electroanalytical performance of electrodes heated at 70 °C for 20 min can be supported by high reproducibility of their basic line across runs and batches, (ESI, Table S4).

The performance of Cu-NPs-modified electrodes used for the detection of UA in fermentation samples was validated by means of the conventional approach, viz. KIT (Uric Acid/Uricase KIT, Table S2). Quantitative results obtained from the intact Cu-NPs-modified electrode were much higher than those defined by the standard Uric acid/Uricase assay, see also (Table S2). This indicates the influence of other electroactive substances present in the samples (yeast and *E. coli* supernatants) on the signal recorded from the intact Cu-NPs-modified electrode. However, after thermal treatment of the same electrode and increasing the amount of Cu_2_O_x_ on the surface of electrodeposited Cu-NPs the amount of UA in fermentation samples was in line with results obtained using conventional uric acid/uricase assay. This demonstrates the increase of specificity of UA determination by electrodes modified with electrodeposited Cu-NPs. However, having the identical specificity of UA analysis in fermentation samples, the proposed amperometric approach is much faster than the fluorescence-based assay (Uric Acid/Uricase KIT), does not require a multi-step sample preparation procedure, and uses a wide range of chemicals.

To conclude, by changes in the surface chemistry of electrodes with electrodeposited Cu-NPs (e.g. by increasing the amount of defect Cu_2_O_x_ and Cu^2+^) it appears to be possible to influence the specificity of UA determination in real fermentation samples at alkaline pH.

## Conclusions

This study explored the electroanalytical performance, e.g., the specificity of electrodes with electrodeposited Cu-NPs in the electrooxidation reaction of UA. The sensing layer containing defect surface copper oxides demonstrated an advanced specificity in UA electrooxidation reactions. The previously reported mechanism of UA oxidation, involving catalysis by Cu^2+^ ions in the presence of molecular oxygen, was used to explain the observed specificity of the electrodes containing defect surface copper oxides. Thus, it was concluded that the catalytic effect of copper(II)-induced oxidation on the surface of electrodeposited Cu-NPs was superior compared to the adsorption stage of UA on copper oxides.

Additionally, the role of oxygen on the surface of electrodeposited Cu-NPs and electrode support material in UA electrooxidation at alkaline pH was highlighted. This observation was important since the oxygen possibly participates in the process of urate ion oxidation by way of a mechanism similar to homogeneous catalysis. In this respect, Cu-NPs containing defect surface copper oxides (Cu_2_O_x_) deposited on the surface of graphene oxide-based electrodes demonstrated superior electroanalytical performance compared with analogs produced on reduced graphene oxide and carbon supports. The design of an electrode sensing layer was optimized and applied for the determination of UA in real fermentation samples.

## Supplementary information

Below is the link to the electronic supplementary material.

ESM 1 (DOCX 1.29 MB)

## Data Availability

No datasets were generated or analysed during the current study.

## Abbreviations

*UA*: Uric acid
*AA*: Ascorbic acid
*Cu-NPs*: Copper nanoparticles
*SPEs*: Screen-printed electrodes
*GO*: Graphene oxide
*r-GO*: Reduced graphene oxide
*SEM*: Scanning electron microscopy
*EDX*: Energy-dispersive X-ray spectroscopy
*GC-MS*: Gas chromatography mass spectrometry
*MSTFA*: N-trimethylsilyl-N-methyl trifluoroacetamide
*OD*: Optical density
*CV*: Cyclic voltammetry
*AM*: Amperometry
*MAM*: Multi-step amperometry
*DFT*: Density functional theory
*Cu*_2_*O*_x_: Defect copper oxides
