# Supplementary material for: Toward the development of a specific non-enzymatic amperometric sensor for determining uric acid in fermentation samples
Source: Mikrochim Acta. 2025 Feb 12;192(3):149. doi: 10.1007/s00604-025-06979-4 (PMC11814019; doi:10.1007/s00604-025-06979-4)
Supplement: Supplementary file 1 — (DOCX 1.29 MB) [file 604_2025_6979_MOESM1_ESM.docx]

**Supplementary Materials**

**Toward the development of a specific non-enzymatic amperometric sensor for determining uric acid in fermentation samples**

*E.V. Butyrskaya^1^, E.V. Zolotukhina^2^, P. Herbeck-Engel^3^, M. Koch^3,4^, Y.E. Silina^5*^*

*^1^Voronezh State University, Department of Analytical chemistry, Voronezh, Russia*

*^2^Federal Research Center of Problems of Chemical Physics and Medicinal Chemistry,*

*Russian Academy of Sciences, Moscow region, Russia*

*^3^INM – Leibniz Institute for New Materials, Saarbrücken, Germany*

*^4^HTW saar – University of Applied Sciences, Saarbrücken, Germany*

*^5^Saarland University, Department of Biochemistry, Saarbrücken, Germany*

**
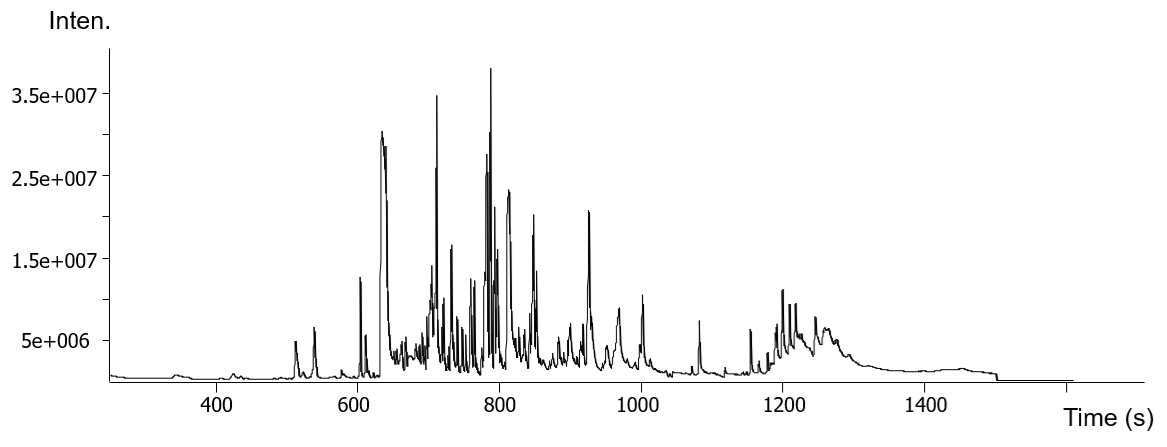
**

**Fig. S1** – GC-MS chromatogram obtained for the yeast supernatant (cultivation time 96 h as a case study) after derivatization with TMSTFA.

***
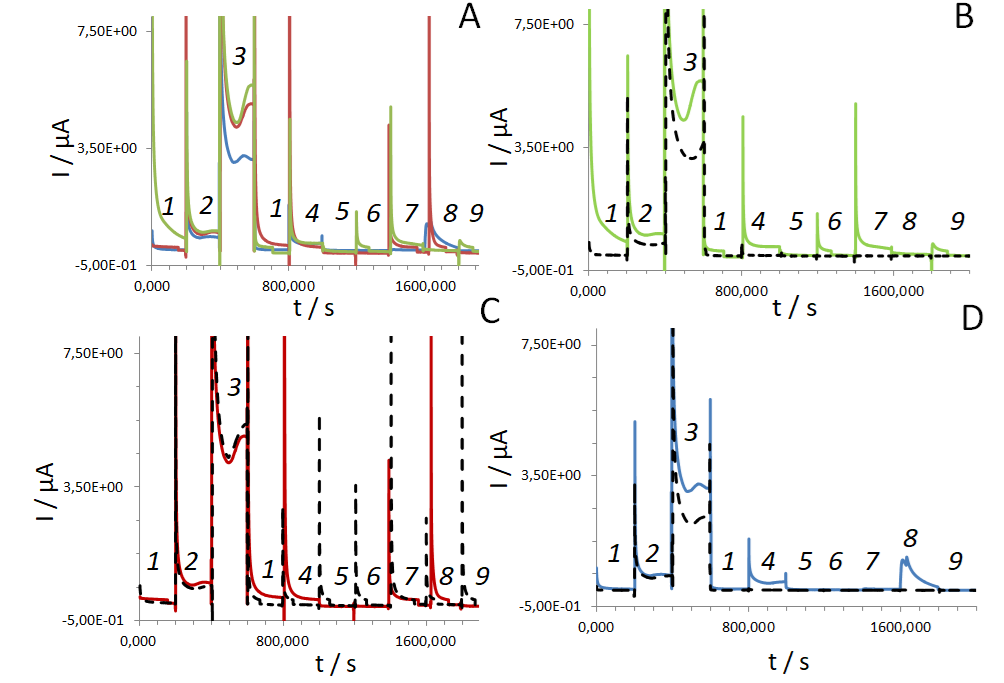
***

**Fig. S2** – AM plots recorded at 0.22 V from Cu-NPs-modified SPEs in: 1 – buffer; 2 – 100 µM UA, 3 – 1 mM UA, 4 – 100 µM AA, 5 – 10 mM glycerol; 6 – 10 mM EtOH, 7 – 10 mM ethylamine; 8 – 10 mM ethanolamine, 9 – 10 mM urea. *Note*: the pH of all tested solutions was 9±0.2. (**A**) – intact electrodes modified with Cu-NPs electrodeposited on: GO (green), r-GO (red), carbon (blue). (**B**) – Cu-NPs deposited on GO before (green) and after heating at 70 °C for 20 min (dashed black line); (**C**) – Cu-NPs deposited on r-GO before (red) and after heating at 70 °C for 20 min (dashed black line); (**D**) – Cu-NPs deposited on carbon before (blue) and after heating at 70 °C for 20 min (dashed black line).

**
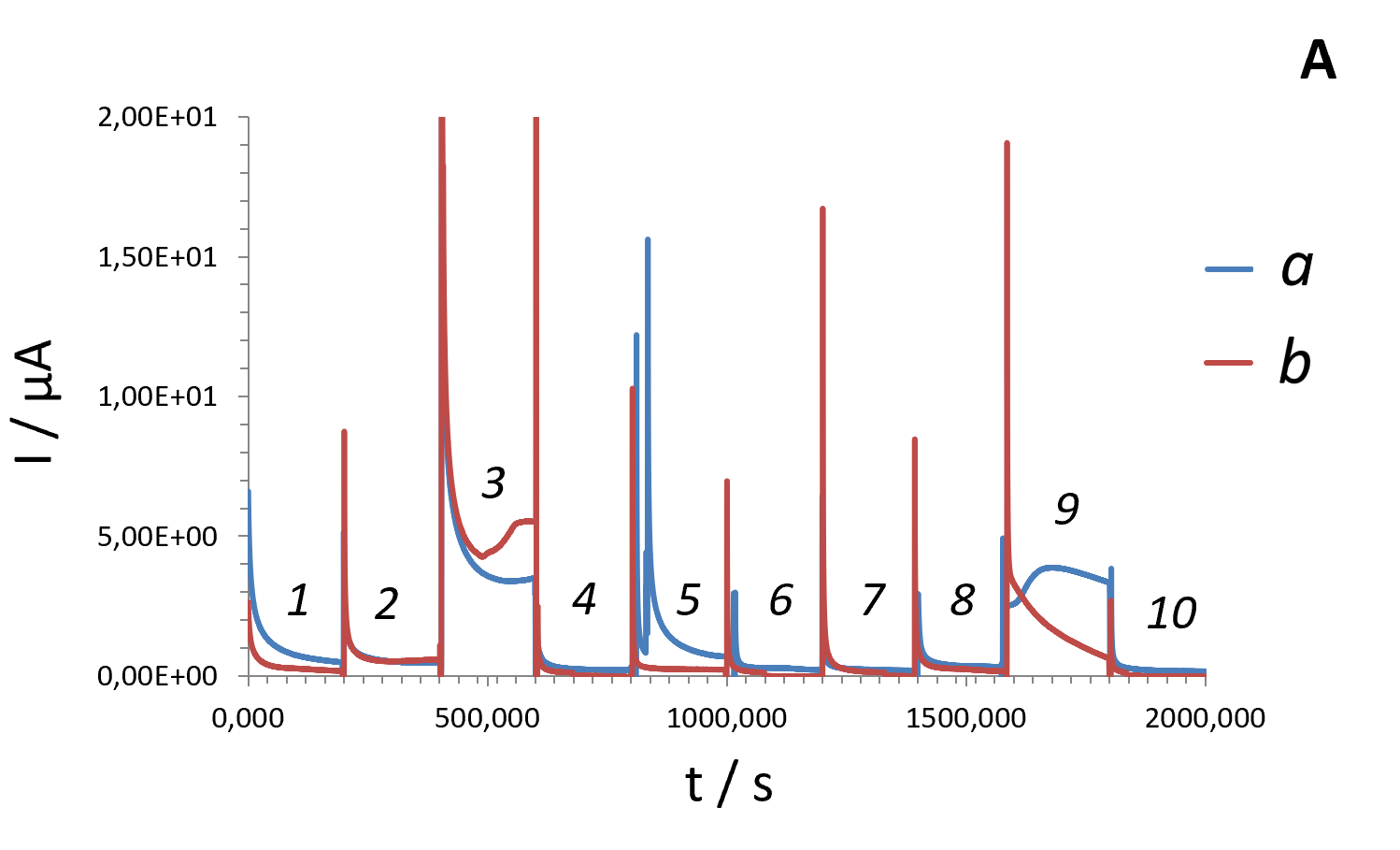
**


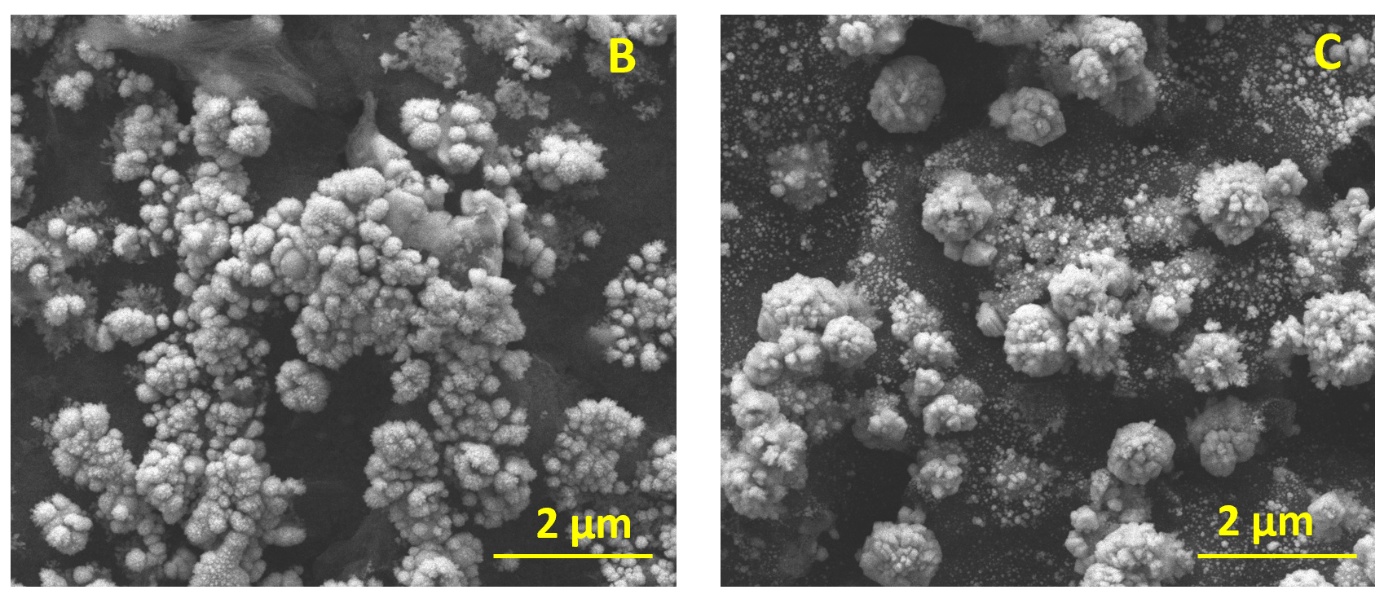


**Fig. S3** – (**A**) AM plots recorded at 0.22 V from larger (100 mM electrolyte) Cu-NPs-modified GO/SPEs in: 1 – buffer; 2 – 100 µM UA, 3 – 1 mM UA, 4 – 100 µM AA (ascorbic acid), 5 – 10 mM glycerol; 6 – 10 mM EtOH, 7 – 10 mM ethylamine; 8 – 10 mM ethanolamine, 9 – 10 mM urea recorded from the intact electrode (*a*) and the same electrode heated at 70 °C (*b*). *Note*: the pH 9 of all tested solutions was 9±0.2. (**B,C**) – SEM images recorded from the intact Cu-NPs-modified GO/SPE (**B**) and from the identical electrode heated at 70 °C and explored during two months (**C**).


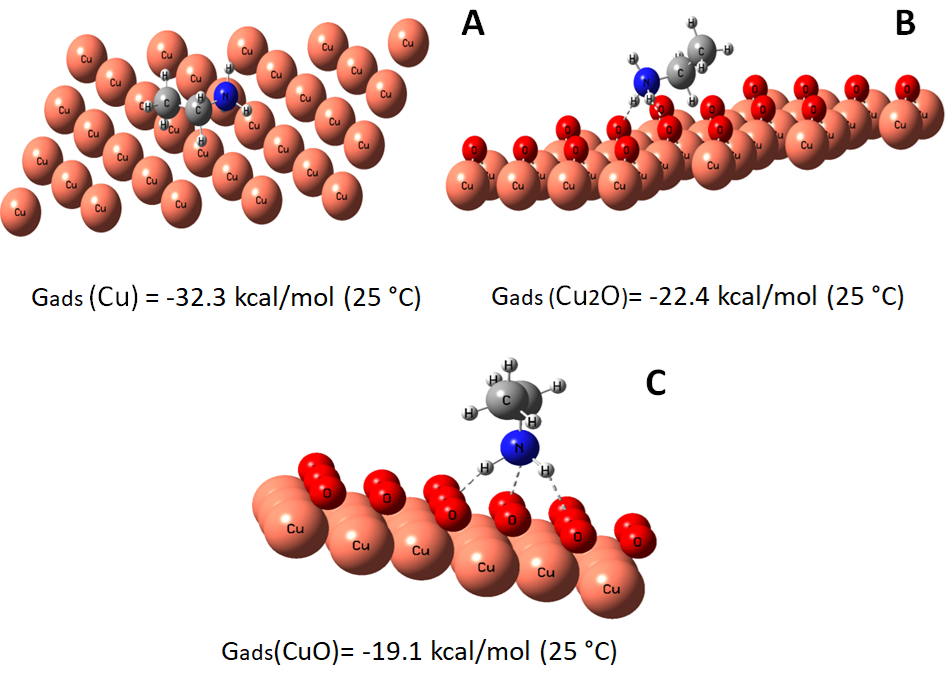


**Fig. S4** – The Gibbs adsorption energies (G_ads_) and the most favorable location of ethylamine on Cu (**A**), Cu_2_O (**B**) and CuO (**C**) surfaces. *Note*: by dashed line the hydrogen bond is shown.


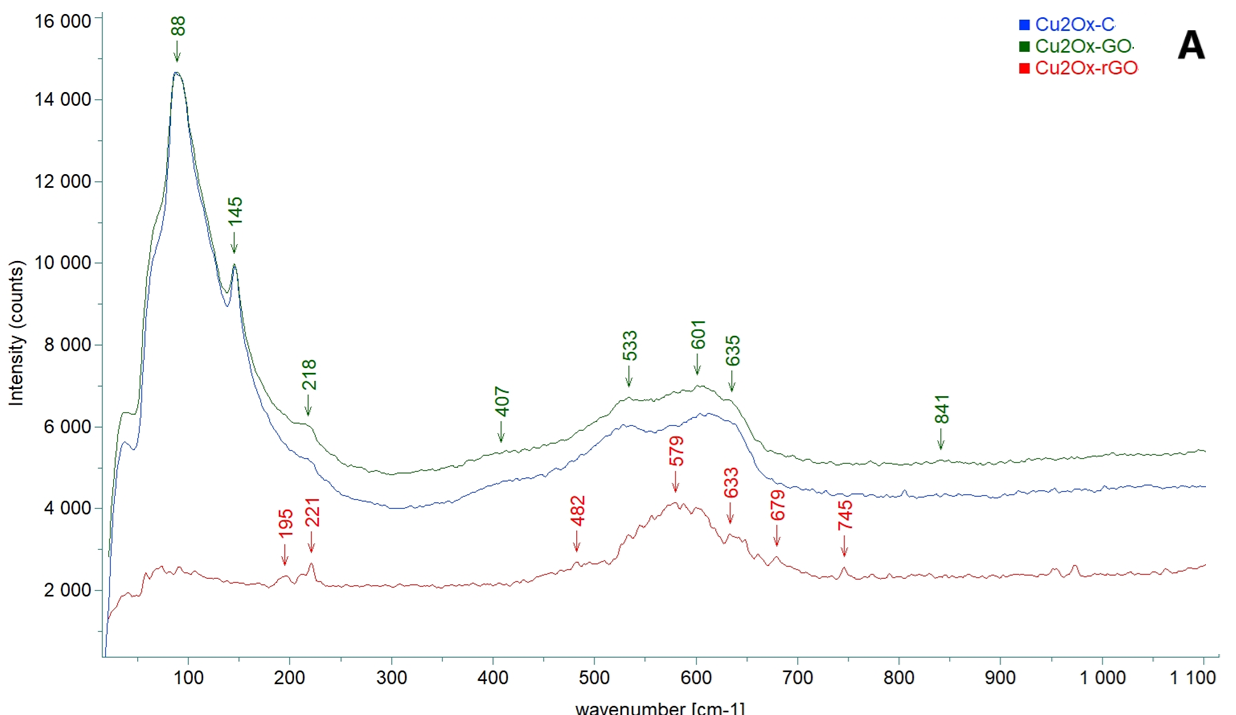


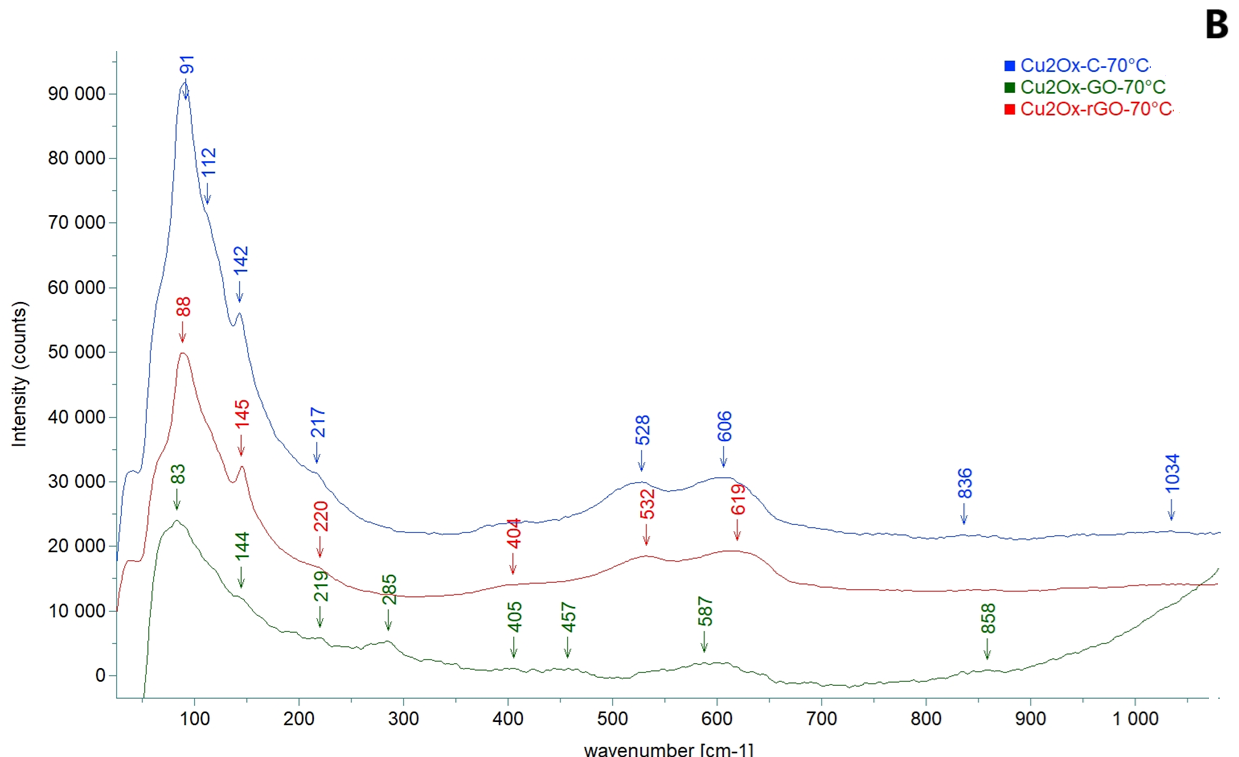


**Fig. S5** – RAMAN spectra recorded from electrodes modified with electrodeposited Cu-NPs (100 mM electrolyte was used as a case study) as prepared (**A**) and after heating at 70 °C for 20 min (**B**). *Note:* c – carbon used as the electrode support material; GO – graphene oxide; r-GO – reduced graphene oxide.


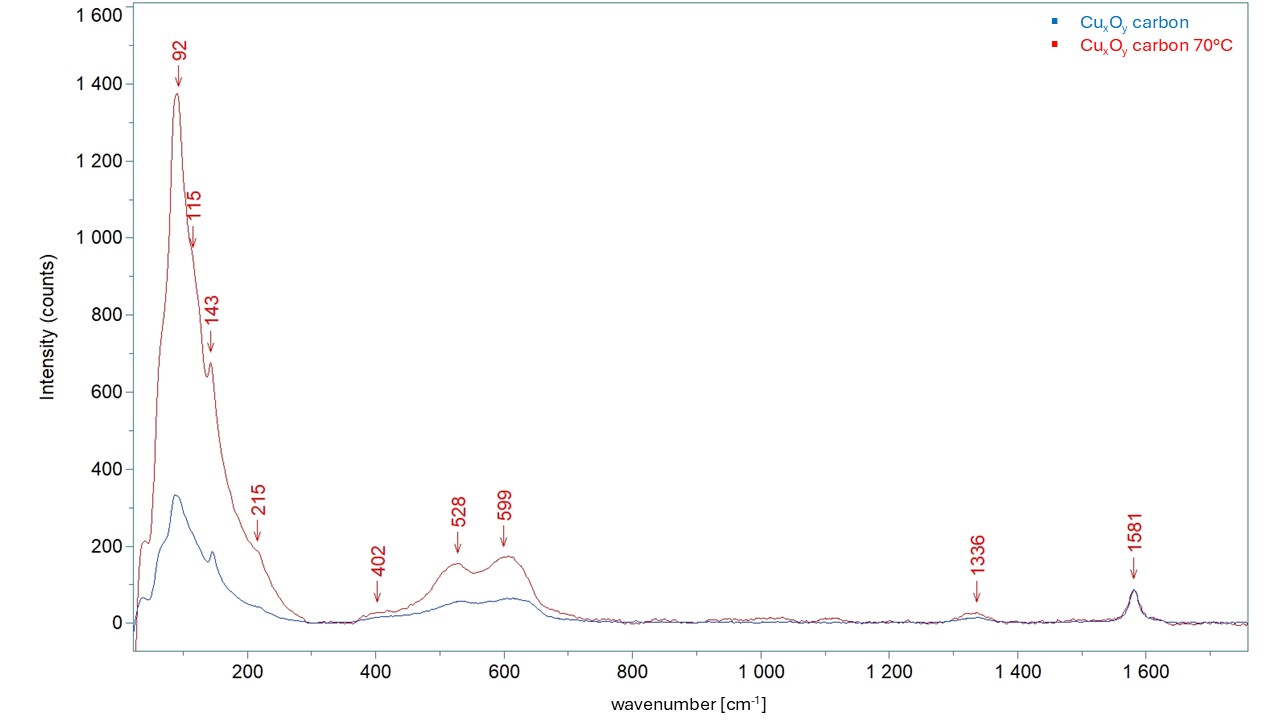


**Fig. S6** – RAMAN spectra recorded from electrodes modified with electrodeposited Cu-NPs (100 mM electrolyte was used as a case study) as prepared and after treatment at 70 °C.

**Table S1** – Qualitative table of selected amines present in supernatants of yeast cells (shown for cells cultivated for 96 h)

| Name | Height | RT(s) | Weight | Unique mass | Quant mass | Quant. S/N^*^ | Peak area |
| --- | --- | --- | --- | --- | --- | --- | --- |
| 1,1,1-trimethyl-silanamine, N,N'-methanetetraylbis | 196502 | 378.8 | 186 | 171 | 171 | 416.3 | 9693405 |
| Tris(trimethylsilyl)amine | 18650679 | 510.2 | 233 | 218 | 218 | 105240 | 549211547 |
| N-Ethyl-4-propyl-4-octanamine | 205802 | 647.6 | 199 | 156 | 156 | 596.4 | 4523817 |
| 1,1,1-trimethyl-N-(1-methyl-2-phenylethyl)-N-(trimethylsilyl)-silanamine | 17272759 | 697.6 | 279 | 188 | 188 | 74522 | 250133093 |
| 2-(p-Chlorophenyl)-ethylamine | 4548 | 750.8 | 155 | 236 | 236 | 29.56 | 106278 |
| 4-ethyl-4H-1,2,4-  Triazol-3-amine | 2978741 | 856.6 | 112 | 112 | 112 | 5784.4 | 48870187 |
| Benzeneethanamine | 18917 | 960.6 | 241 | 446 | 446 | 289.35 | 1338489 |
| Trimethylsilyloxy-methylamine | 7191 | 991.3 | 343 | 345 | 345 | 65.675 | 191586 |
| Adamantanemethylamine | 13916 | 1123.3 | 179 | 190 | 190 | 34.615 | 326441 |

^*^S/N – signal to noise ratio

**Table S2** – The amount (µM) of uric acid (UA) found in tested fermentation samples (supernatants) by conventional fluorescence-based Amplex™ Red Uric Acid/Uricase assay

| Tested  supernatant | Cultivation time of cells, h | Optical density (OD) of cell suspension | Concentration of (UA), µM ±SD | Calibration formula | R^2^ |
| --- | --- | --- | --- | --- | --- |
| *E. coli* | 2 | 2.1 | 28.21±0.01 | y = 1571·x + 7213 | 0.998 |
| *E. coli* | 2 | 1.7 | 10.83±0.03 |  |  |
| *E. coli* | 7 | 3.8 | 3.29±0.05 |  |  |
| *E. coli* | 7 | 4.3 | 3.43±0.05 |  |  |
| *E. coli* | 12 | 6.3 | 0.62±0.09 |  |  |
| Yeast cells | 70 | 5.7 | 1.36±0.03 |  |  |
| Yeast cells | 96 | 6.1 | 0.79±0.02 |  |  |

**Table S3** – Wavenumbers for Cu_2_O, Cu_3_O_4_ and CuO reported in the literature and recorded from the samples in this study (shown for GO/SPE as a case study)

| Literature | [1] | | [2] | | [3] | | [4] | | |
| --- | --- | --- | --- | --- | --- | --- | --- | --- | --- |
| Oxide type | Cu_2_O | CuO | Cu_2_O | CuO | Cu_2_O | CuO | Cu_2_O | Cu_4_O_3_ | CuO |
| Wave numbers [cm^-1^] | 93 |  |  |  |  |  |  |  |  |
|  | 110 |  |  |  |  |  |  |  |  |
|  | 148 |  |  |  | 150 |  | 195 |  |  |
|  | 215 |  | 218 |  | 220 |  | 218 | 218 |  |
|  |  | 296 |  | 298 |  | 297 |  | 285 | 285 |
|  |  | 345 |  | 347 |  | 347 |  |  |  |
|  | 520 |  | 523 |  | 520 |  | 520 | 520 |  |
|  |  |  |  | 591 |  | 605 |  |  |  |
|  | 630 | 630 | 623 |  | 630 |  | 620 | 620 |  |
|  |  |  |  |  |  |  |  | 860 |  |
|  |  |  |  |  |  |  |  | 1135 |  |

| Literature | [5] | | | [6] | | | [7] | | |
| --- | --- | --- | --- | --- | --- | --- | --- | --- | --- |
| Oxide type | Cu_2_O | Cu_4_O_3_ | CuO | Cu_2_O | Cu_4_O_3_ | CuO | Cu_2_O | Cu_4_O_3_ | CuO |
| Wave numbers [cm^-1^] | 93 |  |  |  |  |  | 90 |  |  |
|  | 109 |  |  |  |  |  | 110 |  |  |
|  | 145 |  |  | 150 |  |  | 150 | 175 |  |
|  | 220 |  |  | 220 |  |  | 220 |  |  |
|  |  | 318 | 296 |  | 320 | 300 |  | 280 | 290 |
|  |  |  | 346 |  |  | 350 |  | 320 | 340 |
|  | 520 | 510 |  | 530 | 540 |  | 540 | 530 |  |
|  |  | 540 |  |  |  |  |  |  |  |
|  | 610 | 650 | 631 |  |  |  |  | 610 |  |
|  | 800 |  |  | 650 | 650 | 640 | 650 |  | 620 |

| Literature | [8] | | | [9] | | | [10] | | [11] |
| --- | --- | --- | --- | --- | --- | --- | --- | --- | --- |
| Oxide type | Cu_2_O | Cu_4_O_3_ | CuO | Cu_2_O | Cu_4_O_3_ | CuO | Cu_4_O_3_ | CuO | Cu_2_O  Cu_4_O_3_  CuO _y_ |
| Wavenumbers [cm^-1^] | 90 |  |  |  |  |  |  |  |  |
|  | 110 |  |  | 110 |  |  |  |  | 148 |
|  | 148 |  |  | 140 |  |  |  |  |  |
|  | 218 |  |  | 218 |  |  |  |  | 216 |
|  | 308 | 312 | 298 |  | 312 | 287 | 312 | 290 | 296 / 318 |
|  |  |  | 330 |  |  | 340 |  | 321 | 345 |
|  | 515 | 533 |  | 520 | 515 |  |  |  |  |
|  |  |  |  |  | 534 |  | 515 |  |  |
|  | 612 |  | 602 |  | 627 | 620 | 534 |  | 541 |
|  |  |  |  | 635 |  |  |  |  |  |
|  |  |  |  |  |  |  | 627 | 612 | 651 |

| Oxide type | This work at RT^1^ | | | This work at 70 °C | | | |
| --- | --- | --- | --- | --- | --- | --- | --- |
|  | Cu_2_O on C^2^ | Cu_2_O on GO^3^ | Cu_2_O  Cu_4_O_3_  rGO^4^ | Cu_2_O  Cu_4_O_3_ on C at 70 °C | Cu_2_O  Cu_4_O_3_  GO | Cu_2_O  Cu_4_O_3_  CuO on  rGO | Cu_2_O  Cu_4_O_3_  on rGO |
| wavenumbers [cm^-1^] | 87 | 87 |  | 91 | 90 | 86 | 91 |
|  | 145 | 145 |  | 143 | 143 | 142 | 143 |
|  |  |  | 195 |  |  |  |  |
|  | 218 | 218 | 221 | 216 | 216 |  | 216 |
|  |  |  |  |  |  | 278 |  |
|  |  |  |  |  |  | 322 |  |
|  | 417 | 417 | 483 | 403 | 403 | 450 | 403 |
|  | 528 | 528 |  | 528 | 530 |  | 528 |
|  |  |  |  |  |  |  |  |
|  |  |  | 579 |  |  | 600 |  |
|  | 612 | 612 |  | 606 | 610 |  | 606 |
|  | 638 | 638 | 633 |  |  |  |  |
|  |  |  |  | 836 | 844 | 840 | 836 |

^1^RT – at room temperature (as prepared Cu-NPs);

^2^ – SPE/carbon;

^3^ – SPE/GO

^4^ – SPE/r-GO.

**References:**

1. Levitskii VS, Shapovalov VI, Komlev AE, et al (2015) Raman spectroscopy of copper oxide films deposited by reactive magnetron sputtering. Technical Physics Letters 41:1094–1096. https://doi.org/10.1134/S106378501511022X

2. Henderson E (2010) Lawrence Berkeley National Laboratory (Lbnl). The Military-Industrial Complex and American Society 185–186

3. Zoolfakar AS, Rani RA, Morfa AJ, et al (2014) Nanostructured copper oxide semiconductors: a perspective on materials, synthesis methods and applications. Journal of Materials Chemistry C 2:5247–5270. https://doi.org/10.1039/C4TC00345D

4. Schennach R, Gupper A (2003) Copper Oxidation Studied by In Situ Raman Spectroscopy. MRS Online Proceedings Library 766:32. https://doi.org/10.1557/PROC-766-E3.2

5. Farhad SFU, Webster RF, Cherns D (2018) Electron microscopy and diffraction studies of pulsed laser deposited cuprous oxide thin films grown at low substrate temperatures. Materialia 3:230–238. https://doi.org/https://doi.org/10.1016/j.mtla.2018.08.032

6. Debbichi L, Marco de Lucas MC, Pierson JF, Krüger P (2012) Vibrational Properties of CuO and Cu4O3 from First-Principles Calculations, and Raman and Infrared Spectroscopy. The Journal of Physical Chemistry C 116:10232–10237. https://doi.org/10.1021/jp303096m

7. Meyer BK, Polity A, Reppin D, et al (2012) Binary copper oxide semiconductors: From materials towards devices. physica status solidi (b) 249:1487–1509. https://doi.org/https://doi.org/10.1002/pssb.201248128

8. Purusottam-Reddy B, Sivajee-Ganesh K, Jayanth-Babu K, et al (2015) Microstructure and supercapacitive properties of rf-sputtered copper oxide thin films: influence of O2/Ar ratio. Ionics 21:2319–2328. https://doi.org/10.1007/s11581-015-1403-5

9. Murali DS, Subrahmanyam A (2016) Synthesis of low resistive p type Cu4O3 thin films by DC reactive magnetron sputtering and conversion of Cu4O3 into CuO by laser irradiation. Journal of Physics D: Applied Physics 49:375102. https://doi.org/10.1088/0022-3727/49/37/375102

10. Murali DS, Aryasomayajula S (2018) Thermal conversion of Cu4O3 into CuO and Cu2O and the electrical properties of magnetron sputtered Cu4O3 thin films. Applied Physics A 124:279. https://doi.org/10.1007/s00339-018-1666-6

11. Cocke DL, Schennach R, Hossain MA, et al (2005) The low-temperature thermal oxidation of copper, Cu3O2, and its influence on past and future studies. Vacuum 79:71–83. https://doi.org/https://doi.org/10.1016/j.vacuum.2005.01.010

**Table S4** – Reproducibility of the baseline of electrodes modified with electrodeposited Cu-NPs and heated at 70 °C (evaluated in buffer solution in MAM at pH 9±0.2, read-out at 0.22 V)

| Cu_2_O_x_-NPs/GO  (heated) | Reproducibility of the baseline | | |
| --- | --- | --- | --- |
|  | I, µA | Mean ±SD | RSD, % |
| Run to run | 7.7 ˣ10^-1^  7.1 ˣ10^-1^  6.9 ˣ10^-1^ | 7.23 ˣ10^-1^±0.41 | 5.75 |
| Batch to batch | 7.2 ˣ10^-1^  5.5 ˣ10^-1^  6.2 ˣ10^-1^ | 6.30 ˣ10^-1^±0.85 | 13.56 |
